# Suicide and Coping: Specific Coping Behaviours Associated with Suicidal Ideation and Differences Between Predicted and Actual Coping Among Help-Seeking Individuals

**DOI:** 10.3390/ijerph23060790

**Published:** 2026-06-11

**Authors:** David John Hallford, Emily J. Wallman, Ryan A. Kaplan, Glenn A. Melvin

**Affiliations:** 1 School of Psychology, Deakin University, Geelong, VIC 3220, Australia; 2 Centre for Developmental Psychiatry and Psychology, Department of Psychiatry, School of Clinical Sciences at Monash Health, Monash University, Clayton, VIC 3168, Australia; emily.wallman@monash.edu; 3 Be Psychology & Mental Health, Bondi Junction, NSW 2022, Australia; ryan@be-psychology.com.au; 4 School of Psychology, Faculty of Health, Deakin University, Burwood, VIC 3125, Australia; glenn.melvin@deakin.edu.au; 5 Centre for Educational Development, Appraisal & Research, University of Warwick, Coventry CV1 5FB, UK

**Keywords:** suicide, suicidal ideation, coping, problem-focused coping, help-seeking, emotional distress

## Abstract

**Highlights:**

**Public health relevance—How does this work relate to a public health issue?**

- Suicidal ideation and behaviours are common in communities.
- Gaps exist in our understanding of how people cope and predict they would cope.

**Public health significance—Why is this work of significance to public health?**

- Specific facets of problem-focused coping are used more frequently than other forms of coping.
- Those without a history of suicidal ideation, relative to those with a history, prognosticate more frequent use of adaptive coping strategies.

**Public health implications—What are the key implications or messages for practitioners, policy makers, and/or researchers in public health?**

- Detailed assessment of coping strategies for suicidal ideation may inform intervention and prevention.
- People without lived experience may underestimate challenges with using adaptive strategies.

**Abstract:**

Suicide is a substantial contributor to global mortality, with suicidal ideation (SI) a significant predictor of suicide. Research has demonstrated relationships between dispositional coping styles and SI. This study aimed to advance this research by examining the specific coping strategies people use when experiencing SI. Further, it assessed predicted use of coping strategies of people with a history of SI would differ from the actual coping strategies employed by people have experienced SI. Seventy-seven help-seeking adults (Mage = 31.6, SD = 10.4) with (n = 49) or without (n = 28) history of SI completed the Brief Coping Orientations to Problems Experienced (Brief COPE) adapted to SI-related coping and current emotional distress measured by the 21-item version of the Depression Anxiety Stress Scale (DASS-21). An ANCOVA, while controlling for current emotional distress levels, showed a greater predicted Problem-Focused coping use than actual use reported by participants with SI history. Facet-level ANCOVAs attributed this to differences in Active Coping, Use of Informational Support, and Planning strategies. There were no group differences in emotion-focused or avoidant coping. The preliminary findings suggest individuals without a history of SI may prognosticate more frequent use adaptive coping strategies, relative to how frequently people actually employ them during these times. Future research may examine the factors explaining these differences to help inform programs related to SI and coping.

## 1. Introduction

Suicidal ideation (SI) is a significant predictor of death by suicide, with few other factors, such as previous suicide attempts, demonstrated to be stronger predictors [1]. Additionally, these predictors are interrelated, with SI constituting a prominent reason for psychiatric hospitalisation [2,3], and the presence of SI associated with an increased number of lifetime suicide attempts [4,5]. There is, therefore, value in increasing understanding of SI [2] and its management.

Coping, the cognitive and behavioural efforts and strategies utilised by individuals to manage challenging demands [6,7], is key to an understanding of management of SI. The range of strategies individuals may employ to cope been categorised in numerous ways [7,8]. Broadly, coping strategies may be categorised as either adaptive (i.e., helpful) such as problem-solving and information-seeking, or maladaptive (i.e., harmful) such as substance use and self-pity [9]. Adaptive coping strategies are approach-oriented, and either (e.g., seeking social support actively solving the problem) or (e.g., acceptance, seeking emotional support, or using humour), and involve the individual utiliing their resources and those around them to actively engage with the challenge, in an effort to reduce, eliminate, or counteract their distress [7]. As such, greater use of adaptive coping strategies is associated with positive outcomes such as increased life satisfaction [10]. Conversely, maladaptive coping strategies are avoidant in nature and associated with poorer outcomes [11,12].

Current evidence on coping and suicide has predominantly focused on trait-level coping strategies, that is, the general disposition to use coping strategies in dealing with challenges and how this is associated with suicidal thoughts and behaviours. Within this, greater use of maladaptive coping strategies has been associated with increased SI [9] and suicidal behaviour [13,14]. The association between greater use of avoidant coping strategies and increased SI remains significant after controlling for the influence of prior suicide attempts [15]. Additionally, evidence shows decreased use of adaptive coping strategies to be associated with increased SI [16], while individuals with current suicidality employ less adaptive problem-focused coping strategies than non-suicidal controls [17]. These findings suggest that when experiencing SI, people may fall back on more avoidant and less adaptive forms of coping, potentially due to the emotionally burdening experience and related hopelessness. It may be that the experience of SI degrades adaptive problem-focused coping use over time. Fortunately, some longitudinal evidence shows a reduced likelihood of reporting SI at one-year follow-up among adolescents who more regularly employ adaptive problem-solving coping [18].

The research above highlights how dispositional coping styles correlate with the occurrence of suicidal ideation and behaviour. In addition to a trait differences approach, however, research is needed to identify which specific coping strategies people actually employ in the context of experiencing suicidal thoughts. This would progress understanding from a trait-level analysis of general coping style to a context-specific level, i.e., which strategies do people actually use when suicidal? Another question of interest is how people predict they might cope when experiencing SI. Specifically, whether individuals predicted use of adaptive and maladaptive coping strategies approximates reported by individuals who have experienced SI. Increasing understanding of the relative use of coping strategies when experiencing SI, as well as the similarities and discrepancies in predicted and actual SI-related coping would provide important insight into potential targets for intervention and skill-building and may highlight potential avenues for preventing suicidal behaviour [19].

Consistent with this, the current study had two aims: (1) to investigate the extent of use of different coping strategies in response to suicidal ideation within a help-seeking sample; (2) to examine differences between actual and predicted suicidal ideation coping strategies reported by individuals with and without a history of suicidal ideation. Based on the literature on coping styles, it was hypothesised that those with a history of SI would report less use of problem-focused coping and more use of avoidant coping when experiencing SI, relative to how frequently people who had no prior SI predicted their use of these coping strategies.

## 2. Materials and Methods

### 2.1. Participants

A sample of 77 help-seeking adults (≥18 years) attending private psychology clinics in the states of Victoria and New South Wales in Australia completed an online survey containing the study measures following their first session with a registered clinical psychologist. There were no additional specific eligibility criteria beyond being aged 18 or higher. Participants were categorised into two groups (SI history vs. no SI history) according to whether they indicated a presence of any lifetime history of SI (Have you ever had thoughts about ending your life?) via a dichotomous (Yes/No) item.

A total of 49 (63.6%) participants reported a history of SI (No SI history: *n* = 28, 36.4%). Descriptive characteristics are shown in Table 1. The sample mean age was early 30s, and the majority identified as men. The sample predominantly had undergraduate or postgraduate educational attainment, were in romantic relationships, were overwhelmingly Caucasian, and were in paid work. A quarter were studying at the time of participation.

### 2.2. Materials

Items were administered for participants’ age, gender, ethnicity, relationship status, and education level, and if they were currently studying and/or engaged in paid employment, and to assess their history of SI as noted above.

### 2.3. Brief Coping Orientations to Problems Experienced (Brief COPE *[21]*)

The Brief COPE is a shortened, 28-item version of the COPE Inventory [22] used to assess coping skills utilised by respondents when faced with stressful events. The internal consistency and factor structure of the Brief COPE have been demonstrated to adequately replicate the longer COPE Inventory [21]. Participants indicated the extent to which they employ each of the indicated coping behaviours (e.g., I’ve been making jokes about it) via a 4-point Likert scale (1 = I don’t do this at all, 2 = A little bit, 3 = A medium amount, 4 = I do this a lot). The Brief COPE was modified in this study to probe coping styles specifically related to suicidal ideation. Participants were instructed to think specifically of SI when completing the scale, and individual items were prefaced by the phrase “In relation to suicidal thoughts” to ensure consistency across responses. For participants indicated they had experienced thoughts of ending their life in the past, the scale items referred to how they had actually coped with this. For participants who indicated they had not had thoughts of ending their life, the items referred how they predicted they would cope, including changes to the response scale (e.g., 1 = I would not do this at all). Fourteen specific coping behaviours (known as facets) were assessed, and grouped across three coping subscales: Problem-Focused (facets: Active, Use of nformational upport, Positive eframing, Planning), Emotion-Focused (Six facets: Venting, Humor, Acceptance, Religion, Self-), and Avoidant (Four facets: Self-, Denial, Substance, Behavioral isengagement). Notably, the difference in item instructions means that participants responded to the items in different ways, i.e., to how they actually coped or how they predicted they would cope. This was appropriate given we were primarily interested in whether people who had never experienced SI imagined that they would cope in a similar or different those with experience. However, it is noted that measurement invariance was not tested in this study.

The Brief COPE has shown good psychometric properties at the facet level and at the superordinate, and coping level [23]. Internal consistencies (Cronbach’s α) were acceptable in the current sample at 0.87 (Problem-Focused subscale), 0.64 (Emotion-Focused subscale), and 0.69 (Avoidant subscale).

### 2.4. Depression Anxiety Stress Scale 21-Item Version (DASS-21 *[20]*)

To assess participants’ current emotional distress, the DASS-21 was used, which assesses participants’ levels of core symptoms of depression, anxiety, and stress. Participants respond to 21 brief items (e.g., I felt I was close to panic) via a 4-point Likert scale (0 = Did not apply to me at all; 3 = Applied to me very much, or most of the time) to indicate the applicability of the statement in the preceding week. Total scores range from 063, with higher scores indicating greater emotional distress. The DASS-21 has demonstrated excellent internal consistency in prior research (e.g., total scale Cronbach’s α = 0.93 [24]). The Cronbach’s α for the current sample was 0.95.

### 2.5. Procedure

This study was reviewed and approved by the University Human Research Ethics Committee prior to recruitment. Informed consent was obtained from all participants prior to study participation through the process of sending information to the participants via email after the first session. Where participants were agreeable and consenting, they were then sent a link to the study survey which was hosted on the Qualtrics online survey platform [25]. No compensation was provided.

### 2.6. Data Analysis

The data were analysed using IBM SPSS v.28 [26]. Six paired-sample *t*-tests were conducted to assess differences between coping subscale scores within groups. An independent samples *t*-test was conducted to assess for differences in DASS-21 scores between participant groups. Given that current emotional distress may influence how people retrospectively or prospectively perceive their coping ability [27,28], the DASS-21 total score was entered as a covariate in analyses to control for this influence. Three one-way ANCOVAs were conducted to assess whether presence of lifetime SI history was associated with Brief-COPE subscale scores (Problem-Focused, Emotion-Focused, Avoidant) while controlling for DASS-21 scores. If ANCOVA indicated that SI history status was significantly associated with subscale-level scores a subsequent ANCOVA was conducted to assess for differences between SI history groups on the facets of the indicated subscale while controlling for DASS-21 scores.

## 3. Results

An independent *t*-test showed that participants with a SI history reported significantly greater emotional distress than those without SI history (t [75] = −2.95, d = 0.70, p = 0.004), with these groups reporting scores three and two times higher, respectively, than average scores in the Australian population [29]. Although diagnoses were not systematically recorded as part of the study, almost all participants had been referred through their general practitioner due to significantly impactful mental health issues, most commonly mood, anxiety and personality disorders.

Among participants with SI history, use of reported coping strategies was highest for Problem-Focused, which was significantly higher than for Avoidant strategies, *t*(48) = 2.73, *p* = 0.01, *d* = 0.51. Among participants without an SI history, endorsement of Problem-Focused coping strategies was highest. This was significantly higher than Emotion-Focused, *t*(27) = 6.14, *p* < 0.001, *d* = 1.00 and Avoidant coping, *t*(27) = 5.30, *p* < 0.001, *d* = 1.43. Endorsement of Emotion-Focused coping was also significantly higher than Avoidant coping, *t*(27) = 3.07, *p* = 0.005, *d* = 0.69, for this group. No other significant differences were found (all *p* > 0.05). All Brief COPE scores are displayed in Table 2.

ANCOVA models revealed a significant effect of SI history status on COPE Problem-Focused scores while controlling for emotional distress (*F* [1, 74] = 8.79, *p* = 0.004, η^2^ = 0.11). This indicated that participants without an SI history predicted they would use problem-focused coping strategies if experiencing SI to a significantly greater extent than the reported use of these strategies among people who had experienced SI. There were no significant differences in the Emotion-Focused and Avoidant ANCOVA models (all *p* > 0.05).

Subsequent facet-level ANCOVAs revealed significant between-group differences on Problem-Focused facets of Active Coping (*F* [1, 74] = 6.48, *p* = 0.013; η^2^ = 0.08), Use of Informational Support (*F* [1, 74] = 11.88, *p* = 0.001; η^2^ = 0.13), and Planning (*F* [1, 74] = 6.70, *p* = 0.012; η^2^ = 0.08), but not Positive Reframing (*p* < 0.05). All significant differences indicated greater endorsement of problem-focused coping strategies among participants without SI history than actual use reported among those with SI history.

## 4. Discussion

The current study investigated the use of specific coping styles in relation to suicidal ideation. In addition, the differences between actual coping strategies utilised by individuals with a history of SI and predicted suicide-related coping as indicated by individuals with no history of SI were examined. Findings revealed Problem-Focused to be the most endorsed coping style for SI among participants with SI history, and significantly higher than for Avoidant coping. The findings also demonstrated that the predicted use of problem-focused coping was significantly higher than actual problem-focused coping strategy use by participants with an SI history. Predicted use was higher than actual use for three specific facets (Active Coping, Use of Informational Support, and Planning). No differences were found for emotion-focused or avoidant coping styles.

The finding that problem-focused coping was the most endorsed SI-related coping style among participants with SI history should be viewed in light of prior research demonstrating that use of this coping style is typically reduced among individuals with active suicidality compared to non-suicidal controls [17]. Specifically, the current finding demonstrates that although use of this coping style may be impaired among individuals with a lifetime history of SI, problem-focused strategies still constitute important means of coping with SI. Relatedly, the discrepancy between the actual and predicted problem-focused suicide-related coping reported suggests individuals who have not experienced suicidal thoughts predict that they will respond in problem-focused manner to a stronger degree than the actual use reported by individuals with a history of SI. Such active and approach-oriented coping strategies are generally considered to be adaptive [9,30], which has been specifically demonstrated in relation to SI [17,18]. Therefore, the discrepancy observed in the present study suggests that individuals without a history of SI may be overestimating their ability to cope with potential SI more broadly. Alternatively, higher levels of problem-focused coping in individuals without a history of SI could be protective against the emergence of SI.

The lack of difference between predicted and actual reports of maladaptive avoidant coping is intriguing, particularly considering prior research demonstrating associations between SI and greater use of such strategies [9,15]. Potentially, individuals were able to accurately estimate the extent to which they would employ this style of coping strategy in the event of suicidal ideation. Alternatively, it is possible that individuals with a history of SI under-reported prior use of such maladaptive strategies in the manner of a social desirability bias. This may be due to concerns about possible stigma surrounding some of the specific strategies discussed (e.g., substance use [31]) and would be consistent with the low scores of avoidant coping observed in the current study. Additional investigation incorporating assessment of perceived stigma is required to examine this.

While causal inferences cannot be drawn from these findings, given their cross-sectional nature, they suggest there may be value in improving coping skills among individuals without a history of SI who exhibit risk factors for developing SI (e.g., depression or anxiety [32,33]). The higher endorsement of predicted coping ability relative to actual coping demonstrated in the current study suggests that provision of adaptive coping skills to individuals who are indicated to be at an elevated risk of developing SI is warranted. Providing such skills to at-risk individuals prior to emergence of SI may facilitate effective management of and recovery from SI. However, targeted investigation of preventative coping skill training in terms of change on coping skills specific to SI rather than in general [34], and its impact on SI, is needed. Further, the findings suggest that psychoeducation around SI and coping may be useful, or broader awareness of the concept of safety planning around risk and its effectiveness [35].

Another consideration is why people may draw on these coping strategies in the context of suicidal ideation. This may depend greatly on not only their dispositional coping style, but the cause of mental pain and unfulfilled needs [36]. Indeed, understanding the origins of this mental pain and unfulfilled needs, such as affiliation, autonomy, safety etc., may help establish which coping strategies are useful for people to alleviate or mitigate risk. Further research may explore the phenomenology of SI and how this links to coping strategies that are useful for the individual through that lens., the findings may have relevance to positive psychology interventions that cultivate positive emotions, behaviours, and thoughts/beliefs [37]. Indeed, participants without experience of SI endorsed a likelihood to use such strategies (e.g., positive reframing), indicating perceive merit in these approaches. As noted above, expanded positive psychology preventative interventions may therefore be implicitly acceptable for people, outside of the actual experience of SI. Future research may examine a broader array of positive psychology-related coping (e.g., savoring gratitude) in the context of SI coping.

Regarding study limitations, specific details of prior SI experienced by participants such as severity and recency of ideation were not assessed, and the influence of these factors is unknown. Although all participants had experienced suicidal ideation, itself a predictor of death by suicide [1], understanding how severity impacts coping skills is of note. For example, if the frequency and intensity of suicidal thoughts were high, would this stymie the use of problem-focused copingIn addition, study participants were actively help-seeking, and thus use of specific coping strategies (e.g., ctive oping) may be over-represented due to the help-seeking nature of the sample. Notably these were still not high when considering mean scores on the coping items. A future study examining people with a history of SI but not in therapy would therefore be of use. Future studies might measure additional variables related to coping and SI (e.g., social supportgeneral self-efficacy) to contextualise the use of coping strategies in response to SI. In particular, marrying this strategy-level examination of coping with factors relevant to broader models of suicide, such as thwarted belongingness and perceived burden from the interpersonal theory of suicide [38]. Crucially, how accurate people are at prognosticating how they would cope with SI could be examined through a longitudinal design in which their own actual coping could be assessed, rather than compared to coping of others who had experience SI. Nonetheless, this preliminary study suggests there may be discrepancies. Lastly, the study sample was predominantly Caucasian, highly educated, and exclusively help-seeking in private psychology settings. This should be considered in terms of the generalisability of the findings.

## 5. Conclusions

In conclusion, the current study found that individuals without a history of SI predicted greater use of problem-focused coping strategies in relation to potential SI than actual use reported by individuals with an SI history. These findings suggest that individuals who have not experienced SI more highly endorse the likelihood of coping with SI, relative to actual use of coping, and highlight the value in developing adaptive coping capacity among individuals at risk for developing SI. The current findings provide preliminary but important insights into how individuals cope with SI that can be followed up on in more comprehensive future studies.

## Figures and Tables

**Table 1:** Descriptive characteristics.

	History of SI (*n* = 49)	No History of SI (*n* = 28)	Total Sample (*N* = 77)
	*n* (%)	*n* (%)	*n* (%)
Gender			
*Male*	28 (57.1)	18 (64.3)	46 (59.7)
*Female*	20 (40.8)	10 (35.7)	30 (39)
*Intersex*	1 (2.1)	0 (0)	1 (1.3)
Education			
*Secondary school*	8 (16.3)	2 (7.1)	10 (13)
*Diploma/certificate*	10 (20.4)	2 (7.1)	12 (15.6)
*Undergraduate degree*	18 (36.8)	13 (46.4)	31 (40.3)
*Postgraduate degree*	13 (26.5)	11 (39.4)	24 (31.1)
Relationship Status			
*Single*	25 (51)	7 (25)	32 (41.6)
*Romantic Partner (not cohabiting)*	6 (12.2)	8 (28.6)	14 (18.2)
*Romantic Partner (cohabiting)*	9 (18.4)	9 (32.1)	18 (23.4)
*Married*	9 (18.4)	4 (14.3)	13 (16.8)
Ethnicity			
*Caucasian*	44 (89.8)	28 (100)	72 (93.5)
*Asian*	4 (8.2)	0 (0)	4 (5.2)
*Pakistani*	1 (2)	0 (0)	1 (1.3)
Work			
*Full-time*	26 (53.1)	18 (64.4)	44 (57.1)
*Part-time*	6 (12.2)	6 (21.4)	12 (15.6)
*Casual*	7 (14.3)	2 (7.1)	9 (11.7)
*None*	10 (20.4)	2 (7.1)	12 (15.6)
Study			
*Yes*	14 (28.6)	8 (28.6)	22 (28.6)
Age			
*M* (*SD*)	31.6 (11.6)	31.0 (8.3)	31.3 (10.4)
Range	18–69	20–59	18–69
DASS-21 Score			
*M* (*SD*) Range	29.9 (14.2) 6–63	20.3 (13.3) 0–51	26.4 (14.5) 0–63

Note: SI = Suicidal ideation. DASS-21 = Depression Anxiety Stress Scale [20].

**Table 2:** Brief COPE scores by group.

	History of SI (*n* = 49)	No History of SI (*n* = 28)	Total Sample (*n* = 77)
	*M* (*SD*)	*M* (*SD*)	*M* (*SD*)
**Problem-Focused Coping**	**2.50 (0.60)**	**3.03 (0.68)**	2.69 (0.68)
***Active Coping***	**2.64 (0.80)**	**3.23 (0.74)**	2.86 (0.83)
***Use of Informational Support***	**2.44 (0.85)**	**3.23 (0.91)**	2.73 (0.94)
*Positive Reframing*	2.36 (0.78)	2.54 (0.82)	2.42 (0.79)
***Planning***	**2.55 (0.81)**	**3.13 (0.69)**	2.76 (0.81)
Emotion-Focused Coping	2.34 (0.44)	2.47 (0.40)	2.38 (0.43)
*Emotional Support*	2.66 (0.87)	3.23 (0.81)	2.87 (0.89)
*Venting*	2.32 (0.75)	2.50 (0.83)	2.38 (0.78)
*Humour*	1.96 (0.98)	1.88 (0.99)	1.93 (0.98)
*Acceptance*	2.56 (0.75)	2.52 (0.76)	2.55 (0.75)
*Religion*	1.59 (0.78)	1.98 (0.79)	1.73 (0.80)
*Self-Blame*	2.93 (1.02)	2.70 (0.77)	2.84 (0.94)
Avoidant Coping	2.20 (0.57)	2.13 (0.57)	2.17 (0.57)
*Self-Distraction*	2.88 (0.83)	3.04 (0.68)	2.94 (0.78)
*Denial*	1.96 (0.93)	2.09 (0.95)	2.01 (0.94)
*Substance Use*	2.07 (1.06)	1.82 (0.90)	1.98 (1.00)
*Behavioural Disengagement*	1.90 (0.88)	1.55 (0.82)	1.77 (0.87)

Note: Brief COPE = Brief Coping Orientations to Problems Experienced [21]. SI = ideation. Bolded variables indicate statistically significant differences between the groups.

## Data Availability

The data presented in this study are available on request from the corresponding author in aggregate form. Ethics approval to publicly share participant data was not obtained.
